# Supplementary material for: Cross-border use of health services: An exploratory mixed-methods project at the Mexico-Guatemala border
Source: PLOS Glob Public Health. 2024 Nov 20;4(11):e0003685. doi: 10.1371/journal.pgph.0003685 (PMC11578442; doi:10.1371/journal.pgph.0003685)
Supplement: Attachment — Submitted filename: responsetoreviewers.docx [file pgph.0003685.s003.docx]

Cross-border use of health services: An exploratory mixed-methods project at the Mexico-Guatemala border

**RESPONSE TO REVIEWER**

We appreciate the time the Reviewer dedicated to our manuscript. We provide detailed responses to each comment and recommendation below.

REVIEWER’S COMMENT: This is undoubtedly an interesting paper that demonstrates the cross-border features of the use of health services in two countries belonging to the category of low and middle income countries (LMIC).

RESPONSE: We thank the Reviewer for this comment, we believe this is a relatively underexplored issue, and we are glad the Reviewer found it interesting

REVIEWER’S COMMENT: However, I lacked a bit of data triangulation in this work. Are there any additional or related data that could support the authors' conclusions given the sample size?

RESPONSE: We’re not sure if the Reviewer refers to the mixed-methods study, or just to its qualitative component. The quantitative component was a probability survey with n=4,757 participants, representing 74,673 border crossings. Our results are based on the triangulation of the qualitative data and the results of the probability survey. This is already explained in p. 5 (first paragraph of Methods) but we can add more detail if the Reviewer thinks it’s needed.

REVIEWER’S COMMENT: In addition, the work would have been richer if the authors had briefly described the characteristics of the health care system of both countries. It also affects the search for services in a neighboring country.

RESPONSE: We added a description of the health systems of both countries in the Introduction (“The health systems of both countries include...”, pp. 4-5). We also moved a phrase that previously appeared on the Methods section (“...the Mexico-Guatemala border region, a zone of intense social and economic exchange between the two countries”) to this paragraph, so the paragraph presents the context of the study before moving into the study’s aims.

REVIEWER’S COMMENT: Furthermore, I think it would be useful and also interesting to illustrate the example of other countries to compare the findings and trends. The work just only briefly mentions the example of Brazil

RESPONSE: There is very little published about this subject, but following the Reviewer’s recommendation we added comparisons to the results of studies in the Dominican Republic – Haiti and Laos – Thailand borders (p. 23).

I am asked about the availability of all data, and the authors indicate the following:
"The quantitative data used for this article will be made publicly available in the first author's institutional repository. The qualitative data contains sensitive information and therefore cannot be shared." However, I did not see any link or redirect to access the quantitative data. And looking for the repository of the first author's institution takes time. I may have missed it somewhere, so it would be helpful if this information was somehow presented in a way that would not take time to search for it further.

RESPONSE: The dataset hasn’t been given clearance for publishing yet, so we can`t provide a link.
